# PupEyes: An interactive Python library for eye movement data processing

**DOI:** 10.3758/s13428-025-02830-z

**Published:** 2026-01-05

**Authors:** Han Zhang, John Jonides

**Affiliations:** University of Michigan, Ann Arbor, MI USA

**Keywords:** Eye-tracking, Pupillometry, Fixation, Python, Data analysis

## Abstract

We present PupEyes, an open-source Python package for preprocessing and visualizing pupil size and fixation data. PupEyes supports data collected from EyeLink and Tobii eye-trackers as well as any generic dataset that conforms to minimal formatting standards. Developed with current best practices, PupEyes provides a comprehensive pupil preprocessing pipeline and interactive tools for data exploration and diagnosis. In addition to pupil size data, PupEyes provides interactive tools for visualizing fixation data, drawing areas of interest (AOIs), and computing AOI-based metrics. PupEyes uses the *pandas* data structure and can work seamlessly with other data analysis packages within the Python ecosystem. Overall, PupEyes (1) ensures that pupil size data are preprocessed in a principled, transparent, and reproducible manner, (2) helps researchers better understand their data through interactive visualizations, and (3) enables flexible extensions for further analysis tailored to specific research goals. To ensure computational reproducibility, we provide detailed, executable tutorials (https://pupeyes.readthedocs.io/) that allow users to reproduce and modify the code examples in a virtual environment.

Researchers have long been interested in the relationship between eye movements and mental processes (Javal, 1878). Eye movements such as fixations and saccades offer valuable insights into an observer’s moment-to-moment cognitive processing. In addition to tracking gaze position, most modern eye-trackers also record pupil size, a physiological measure associated with a wide range of cognitive processes (Mathôt, 2018) and the neurobiology of the locus coeruleus (Aston-Jones & Cohen, 2005). In recent years, eye-tracking has become increasingly accessible, partly due to the growing popularity of open-source tools. Originally developed as a general-purpose language, Python is now extensively used for scientific computing and data analysis. Within psychology, Python-based tools such as OpenSesame (Mathôt et al., 2012) and PsychoPy (Peirce, 2007) have gained traction for experimental design. These programs are compatible with many modern eye-tracking systems, which now offer an open-access, low-level application programming interface (API), allowing researchers to conduct eye-tracking experiments without purchasing commercial software. Furthermore, data analysis libraries such as pandas (McKinney, 2010; The pandas development team, 2020) and MNE (Gramfort et al., 2013) make it convenient, and sometimes necessary, to design experiments and analyze data within a unified Python environment.

With the growing accessibility of eye-tracking technology comes a considerable variability in how pupil size and fixation data are processed. While several recent reviews have outlined best practices for pupil data analysis (e.g., Kret & Sjak-Shie, 2019; Mathôt & Vilotijević, 2022; Mathôt et al., 2018; Reilly et al., 2024; Steinhauer et al., 2022; Winn et al., 2018), there has been limited effort to implement these practices in Python. Moreover, in our experience, one of the most critical aspects of eye movement data analysis is effective data visualization. Human observers make rapid, moment-to-moment decisions about where and when to look. Being able to “relive” these decisions through visualization helps researchers better understand their data and uncover new insights. Visualization is also critical in pupil data preprocessing, which typically involves applying multiple user-defined preprocessing steps to high-temporal-resolution signals. As open-source eye-tracking becomes more popular, an unintended consequence is that analyses are often carried out entirely in tabular form without easy access to data visualization, a feature commonly provided by proprietary software. Researchers need a simple and flexible way to inspect data quality at any stage of the preprocessing pipeline for any individual trial. Unfortunately, such visualizations are still rare in existing open-source packages for eye movement data analysis.

Here, we present PupEyes, an open-source Python package for preprocessing and visualizing pupil size and fixation data. Key features of PupEyes include the following:

- **Best practices:** The pupil preprocessing functions are developed based on current best practices from the literature. Using PupEyes ensures that pupil size data is preprocessed in a principled, transparent, and reproducible manner.
- **Interactive visualizations:** PupEyes provides multiple interactive visualizations that enable researchers to explore their data, inspect individual trials, and identify potential outliers.
- **Pandas integration:** PupEyes uses the pandas data structure, giving researchers full access to powerful tools for data manipulation, analysis, and visualization within the broader Python ecosystem.

While PupEyes provides interactive visualizations, it is designed not to be purely a graphical user interface (GUI) application. As others have noted, a pure GUI is accessible to beginners but often comes at the cost of reproducibility (Geller et al., 2020). Furthermore, experienced users often prefer the freedom to perform custom analyses without being limited to a fixed set of predefined operations. Using PupEyes requires some basic familiarity with the Python programming language. In particular, we encourage users to become familiar with pandas (McKinney, 2010; The pandas development team, 2020), one of the most widely used Python libraries for data analysis and manipulation. Integrating with pandas offers several advantages. For one, pandas provides a rich set of efficient and well-documented tools for data analysis and manipulation. It also integrates seamlessly with other commonly used Python packages in machine learning, statistics, and data visualization. From a trainee’s perspective, knowing Python, particularly pandas, is a highly valuable and transferable skill.

PupEyes is also not a “one-click” solution for data processing. Because pupil size data vary widely in collection methods and research goals, a single pipeline rarely fits all datasets. Instead, PupEyes provides a comprehensive toolkit for researchers to customize processing pipelines according to their specific needs.

The remainder of the paper will provide a hands-on guide to PupEyes’ core functionalities, with detailed code examples and explanations.

## Package installation

PupEyes requires Python 3.10 or above. To install PupEyes, run the following from the command line:
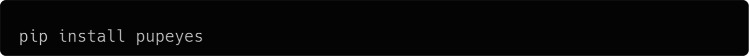


Users can install the latest development version of PupEyes by running the following instead:
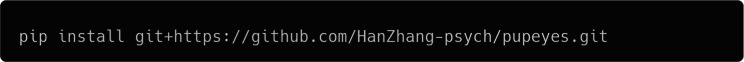


We recommend using PupEyes in Jupyter Notebook (Pérez & Granger, 2007), a web-based interactive computing interface, to take full advantage of PupEyes’ features. For more information on installing and using Jupyter Notebook, please see https://jupyter.org/. PupEyes has detailed tutorials (https://pupeyes.readthedocs.io/). To ensure computational reproducibility, the above tutorials are executable in a virtual environment. To launch the executable tutorials, users can click the “Try PupEyes in MyBinder!” link on the home page or the rocket icon on any specific tutorial page. This will launch a virtual Jupyter Notebook server, where users can reproduce and modify the codes without having to install PupEyes locally.

## Pupil size preprocessing

**Fig. 1:**
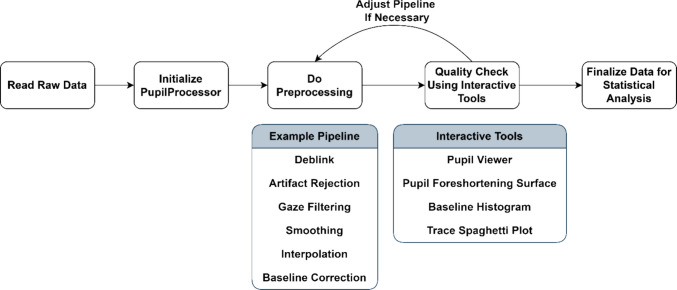
A flowchart of typical steps needed for pupil size preprocessing using PupEyes

Figure 1 summarizes the typical steps of pupil size preprocessing using PupEyes.

**Fig. 2:**
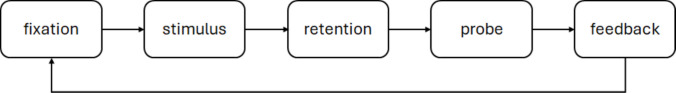
A flowchart of the sequence of events in a single trial of the example experiment

To illustrate these steps, we will use a simple memory task as an example. On each trial, participants were first shown a series of X’s at the center of the screen on which to fixate. Then, the X’s were replaced by a string of letters (e.g., APIWV) that participants were asked to remember. After displaying letters, the screen returned to a series of X’s to mask the stimulus. After a while, a single letter appeared on the screen (e.g., P), and participants were asked to indicate whether the letter belonged to the memory set. Once a response was made, participants received feedback about their accuracy. The sequence of events is summarized in Fig. 2.

### Read raw data

**Fig. 3:**
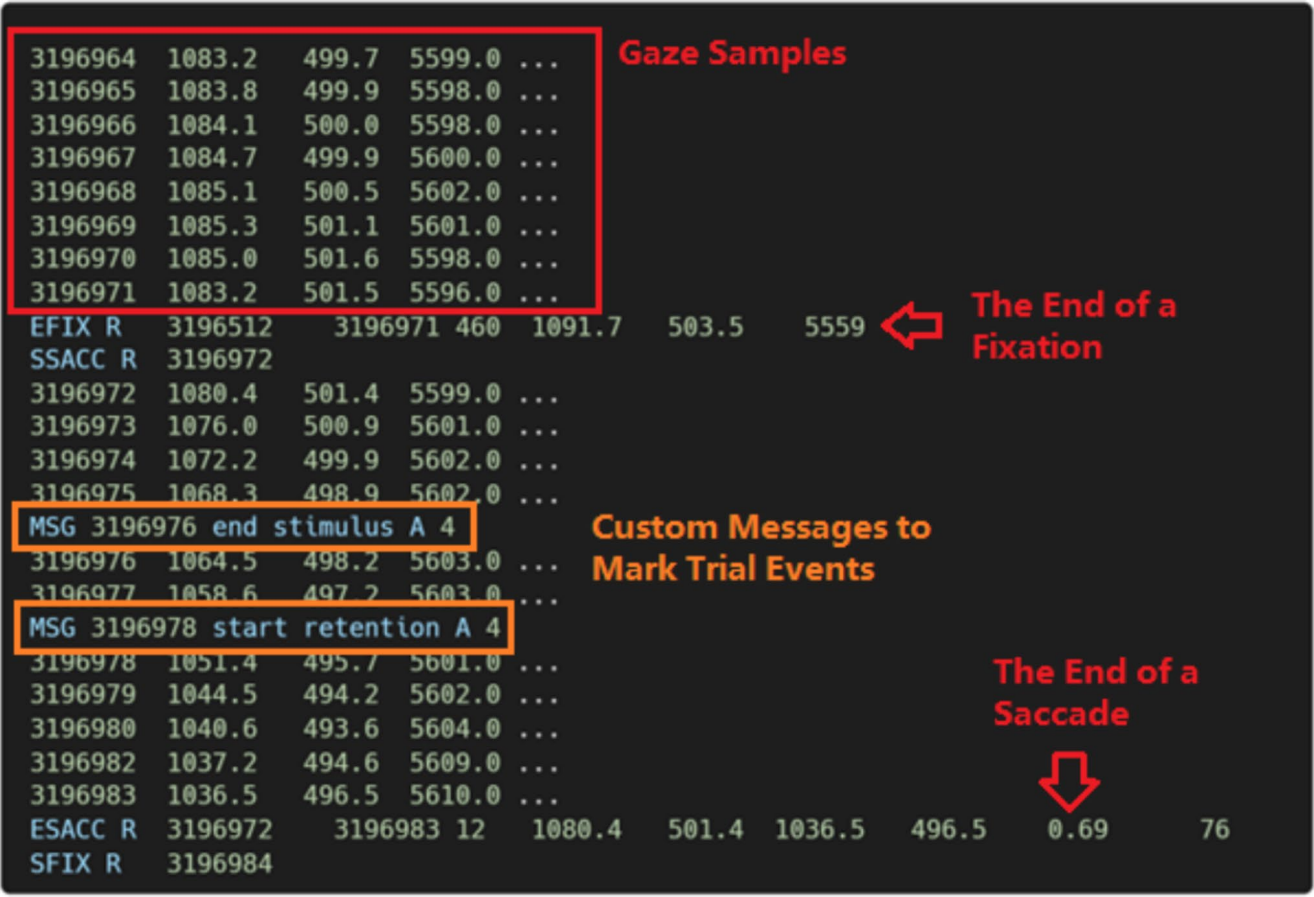
An excerpt of EyeLink .asc data. Rows starting with “MSG” indicate custom messages that mark events during a trial

For pupil preprocessing, PupEyes expects a pandas data frame with basic columns such as timestamps, x and y coordinates, pupil size, and associated event messages. Data from any eye-tracker can be used as long as the required information is included. Some eye-tracking systems, such as the popular EyeLink system (SR Research Ltd.), save data in raw format that requires significant data wrangling before it can be used. Here, we use PupEyes to read raw data collected with an EyeLink 1000 Plus eye-tracker. EyeLink saves data in EDF format, and these files need to be converted to ASC format before PupEyes can read them. This conversion can be easily done (www.sr-research.com/support/thread-7674.html). Figure 3 shows an excerpt of an EyeLink data file.

Rows starting with “MSG” are custom messages that mark the start or end of trial events. These messages typically follow a fixed format with dynamically adjusted values. For example, “start fixation A 4” marks the start of the event “fixation” on block A trial 4. PupEyes looks for these MSG rows to read trial data. Specifically, the following information must be specified:

- **Filename.** PupEyes reads EyeLink .*asc* data converted from the original .*edf* format.
- **Event marker format.** In our case, the event markers consist of four parts: marker (a string), event (a string), block (a string), and trial (an integer).
- **Delimiter.** The delimiter for the event marker message. In our case, it is a space.
- **Start and stop messages.** The messages that define a trial’s boundary. In our case, a trial always starts with *start fixation* and ends with *end feedback*. PupEyes will read data within the trial boundary.
- **Constant columns (optional)**. Users can specify custom columns that take a single value (e.g., participant identifier) to include in the parsed data.

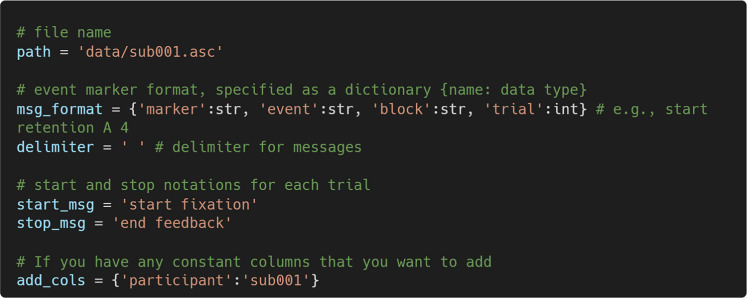


**Fig. 4:**
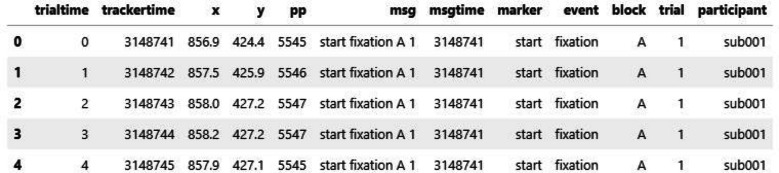
Example gaze data extracted by PupEyes

Then, we can create an EyelinkReader object by passing the required information:
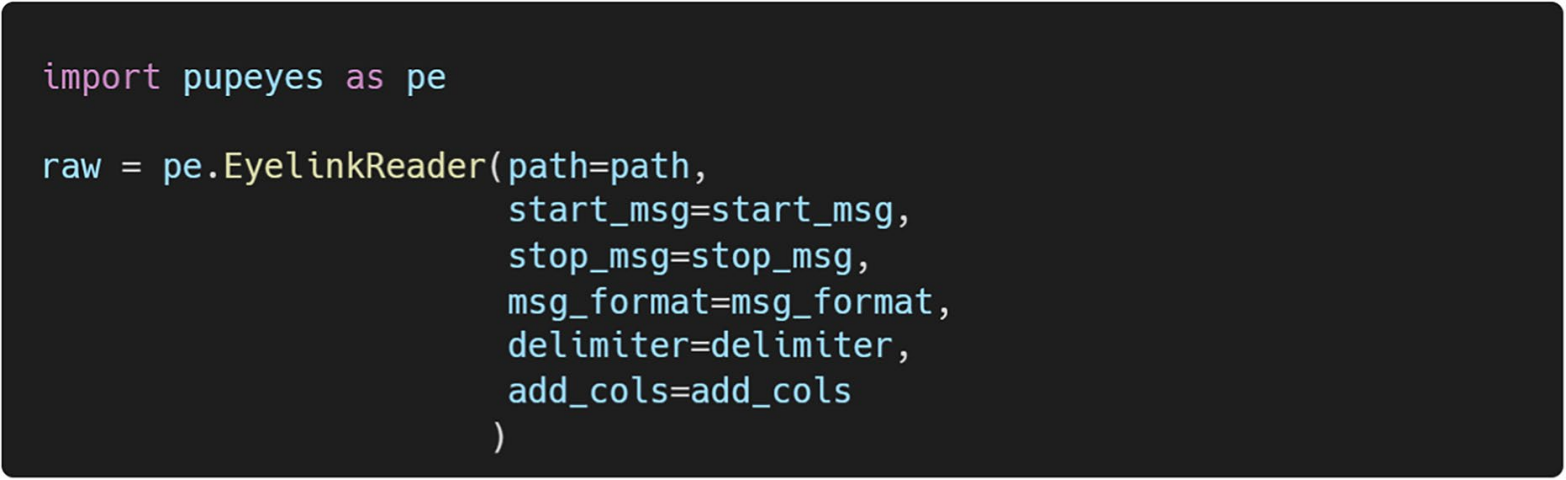


Once an EyelinkReader object is created, we can easily extract gaze samples, fixations, saccades, and blinks:
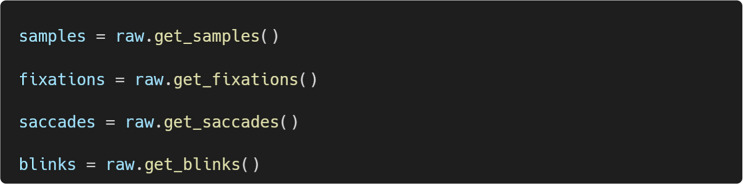


Figure 4 shows the first five rows of gaze samples extracted using the get_samples() method. Each row represents a single gaze sample, collected at a sampling rate of 1,000 Hz. Each sample is paired with the most recent event marker message in the data file, parsed according to the user’s specification. Specifically, trialtime indicates time in milliseconds within a single trial, and trackertime reflects time in milliseconds on the system clock. x and y are gaze coordinates, and pp represents pupil size. msg and msgtime store original messages and their corresponding system timestamps. Constant columns are added to the data file if specified.

When designing an experiment, researchers must decide where an event marker is necessary and how it should be formatted. The specific method for sending these messages varies depending on the experiment-design environment and is beyond the scope of this paper. However, relevant information can be found online (e.g., OpenSesame: https://osdoc.cogsci.nl/4.0/tutorials/visual-world/, PsychoPy: https://psychopy.org/api/iohub/starting.html, Pylink: https://www.sr-research.com/support/thread-48.html). When there are multiple events within each trial, marking the start and end of each event can provide flexibility in data parsing. For example, one can specify a different trial boundary to extract data for part of a trial.

### Reading Tobii data

PupEyes also supports reading data from Tobii eye-trackers. Traditionally, it is common practice for Tobii users to run experiments using Tobii Pro Lab and export the data for customized analyses. However, it is also possible to use the Tobii Pro SDK to capture data streams when running PsychoPy or MATLAB experiments directly. For example, Titta (Niehorster et al., 2020) is a toolbox allowing Tobii eye-trackers to interface with PsychoPy (Peirce, 2007) experiments. Titta saves data streams in HDF5 format. PupEyes supports reading data from both Tobii Pro Lab and Titta. Tobii users are recommended to review the online tutorial (https://pupeyes.readthedocs.io/en/latest/tobii_data.html) for specific instructions.

### Reading data from multiple participants

When reading data from multiple participants, PupEyes can be combined with standard Python syntax to iteratively read each participant’s data and combine them into a single pandas data frame. For example, the code below uses a “for” loop to extract fixation data for each participant in the same directory and combines them into a single data frame.
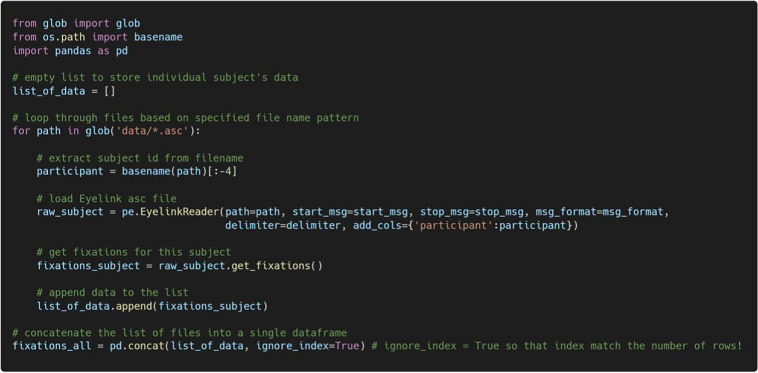


### Converting to trial-based format

As shown in Fig. 4, PupEyes uses a long format in which each row represents a single gaze sample. While intuitive, this format can produce very large datasets, and some columns (e.g., trial ID, block, condition) remain constant within a trial. For this reason, some packages opted to use a wide (i.e., trial-based) format, where each row represents one trial with pupil sizes for that trial stored as a list. If users wish to work with packages that require a trial-based format, we provide an example in the online tutorial for format conversion (https://pupeyes.readthedocs.io/en/latest/read_data_eyelink.html#convert-to-trial-based-format). For all analyses introduced below, however, the data are represented in the long format.

### Initialize PupilProcessor

PupEyes performs all pupil preprocessing steps through a PupilProcessor object. This object is initialized by providing properly formatted gaze data. Data extracted using the get_samples() method already conforms to the formatting requirements (see Fig. 4). Any generic eye-tracking dataset can work as long as it has column(s) that identify a unique trial, a timestamp column, x and y coordinates, and pupil size.

In the example below, we create a PupilProcessor object named p.
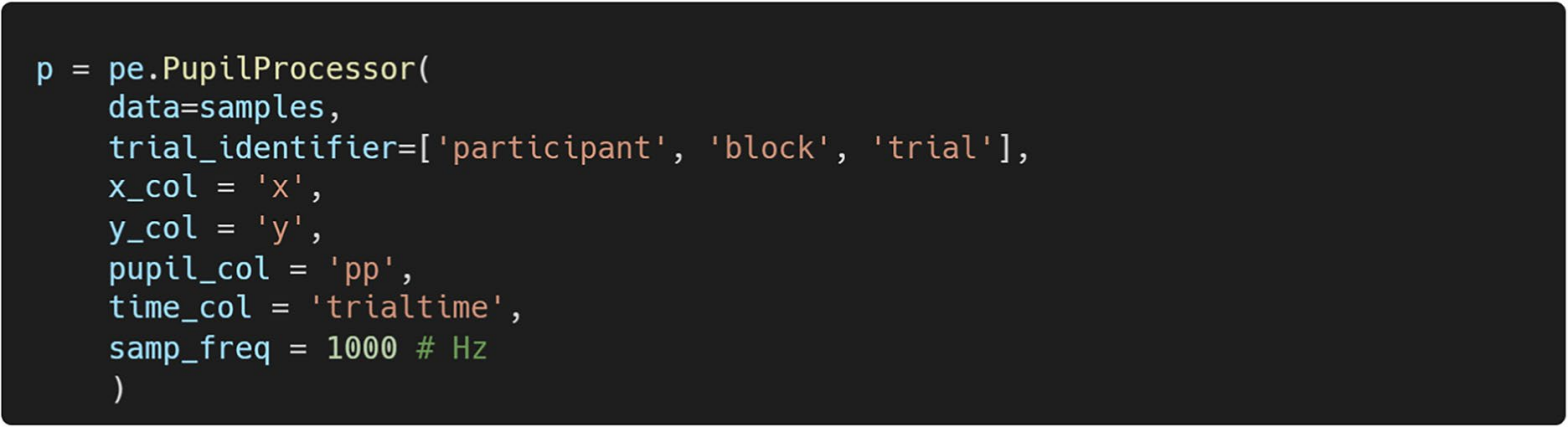


trial_identifier represents the column(s) that differentiate one trial from another in the data. Pupil preprocessing is performed separately for groups defined by trial_identifier. This also means that PupEyes inherently supports processing multiple participants in one instance as long as trial_identifier is specified correctly.

### Do preprocessing

Once a PupilProcessor object is created, we can perform preprocessing using the available methods. The preprocessing pipeline introduced here serves as an example and does not cover all pupil preprocessing features provided by PupEyes. Researchers should refer to the detailed tutorials (https://pupeyes.readthedocs.io/) and design their own preprocessing pipeline suitable for their data.

#### Deblink

Deblinking involves identifying samples during blinks and setting them as missing. During a blink, the eye-tracker temporarily loses the pupil signal. Many systems, such as EyeLink, record missing pupils as 0. In addition to a complete loss of signal, the recorded pupil area is also distorted by the opening and closing of eyelids. These reasons make deblinking a critical preprocessing step. PupEyes uses a blink detection algorithm developed by Hershman et al. (2018), which effectively removes samples during blinks and samples during the opening and closing of eyelids.

Running deblinking requires just one line of code.
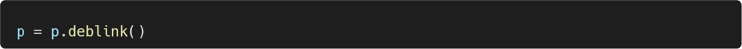



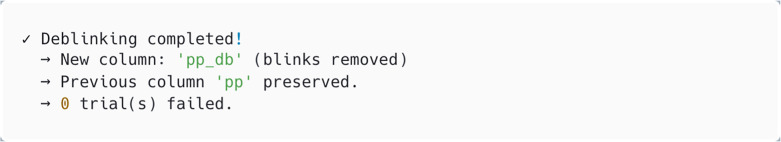


For every processing step performed, PupEyes adds a new column with a suffix to store the new data while preserving the existing data. The printed message informs the user which column is created for which specific operation. The resulting pupil data are stored as a pandas data frame and can be accessed through the .data attribute.
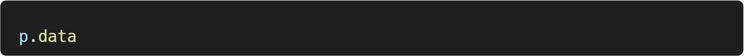


**Fig. 5:**
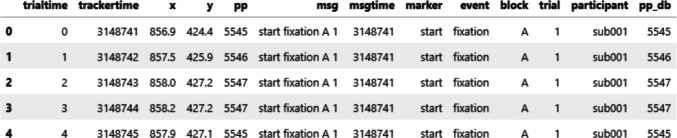
Data after deblinking. A new column “pp_db” is created to indicate deblinked pupil sizes

The resulting data frame is shown in Fig. 5. Here, column *pp* shows the original pupil data, and column *pp_db* shows the deblinked data.

#### Resampling (optional)

Many modern eye-trackers record pupil data at very high rates (e.g., 1,000 Hz), which may be excessive given the slow nature of pupillary responses. To speed up processing, users may choose to downsample to a lower frequency. However, some preprocessing steps, such as deblinking, benefit from high-frequency data, so it is recommended that downsampling be undertaken after removing blinks (Mathôt & Vilotijević, 2022). Please refer to the online tutorial for instructions on downsampling (https://pupeyes.readthedocs.io/en/latest/pupil_preproc.html#downsampling-optional).

In other cases, the user may want to upsample the pupil data to match the sampling rate of other devices (e.g., electroencephalography). PupEyes also provides a function for upsampling the data (https://pupeyes.readthedocs.io/en/latest/api.html#pupeyes.pupil.PupilProcessor.upsample). The latter steps in this paper are performed without resampling.

#### Artifact rejection

In addition to blinks, other factors such as sudden head movements, squinting, or an eye-tracker malfunction can also result in inaccurate pupil measurements. Moreover, if some blinks are not successfully detected during the initial deblinking step, further cleaning may be required to address the remaining artifacts. Therefore, it is often necessary to perform additional data cleaning after deblinking. PupEyes implements an *artifact_rejection()* method for this purpose. Users can opt to remove pupil size artifacts using one or both methods:

- **Rapid change in pupil size.** This method identifies rapid changes in pupil size and has been used in several existing packages (Geller et al., 2020; Kret & Sjak-Shie, 2019; Tsukahara, 2022). First, the method computes pupil speeds, defined as the absolute maximum of the forward and backward differences at each point, normalized by the time difference. Then, a speed threshold is set as [Median pupil speed + *n* × MAD], where MAD is the median absolute deviation of pupil speeds. The default value of *n* is 16 (Kret & Sjak-Shie, 2019). Pupil measurements with a speed value above the threshold are set to missing.
- **Extreme pupil sizes.** This method identifies extreme pupil sizes using *z*-scores (Dalmaijer et al., 2014). Pupil sizes are set to missing if they exceed a *z*-score threshold (default = 2.5), but only when the pupil trace’s coefficient of variation (sd/mean) exceeds a certain value (default = 0.1). Using the coefficient of variation threshold protects stable data from being removed.

The default is to use both methods, but the user may choose to use only one by specifying *method*='*speed’* or *method='zscore’*. Here, we use both methods for artifact rejection.
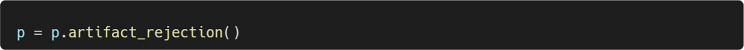


#### Filtering gaze positions

Many pupillometry experiments present stimuli at the screen center for a good reason: to minimize pupil foreshortening error (PFE). PFE refers to an artifact in which measured pupil size is reduced due to the eyes rotating away from the camera, causing the measured pupil area to shift from a circle to an ellipse. However, completely eliminating PFE is unrealistic, as participants are unlikely to maintain fixation on a single point throughout an entire study. Furthermore, certain tasks, such as reading or scene perception, naturally require eye movements. Although algorithmic methods exist to correct PFE during data analysis (e.g., Hayes & Petrov, 2016), these corrections may inadvertently distort the data if the underlying model is inaccurate (Kinley & Levy, 2021). It is recommended that, at a minimum, researchers should ensure that there is no confound between eye position and experimental conditions (Mathôt & Vilotijević, 2022). In line with this recommendation, PupEyes allows users to define a valid region for pupil measurement. Any samples falling outside this region are set as missing. This position-based filtering can help mitigate PFE by limiting analyzed samples to a consistent region.

For demonstration purposes, we set the valid region to the screen area. In a later section, we introduce an interactive diagnostic plot that allows users to examine the extent of PFE in their data.
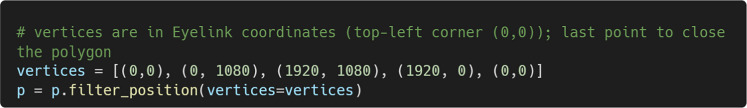


#### Smoothing

Eye-trackers typically produce high-frequency noise that should be smoothed out before analysis. Here, we will use the default option: A Hann window applied over 100 *samples* (i.e., 10 ms at a 1,000 Hz sampling rate). PupEyes also offers two alternative smoothing methods: a rolling mean and a Butterworth low-pass filter.
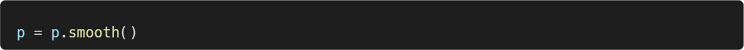


#### Interpolation

So far, the preprocessing steps have set inaccurate pupil measurements as missing. It may be desirable to interpolate these missing values to obtain a more continuous pupil trace. PupEyes supports two commonly used interpolation methods: linear interpolation and cubic-spline interpolation.

Linear interpolation draws a straight line connecting the endpoints of a gap in the data. This method is simple and computationally efficient; however, the resulting interpolated values may not resemble a natural pupil trace. In contrast, cubic-spline interpolation generates a smoother curve that more closely resembles a natural pupil trace, but it is computationally more extensive and may occasionally introduce artifacts. Therefore, it is important to carefully inspect the interpolated values to ensure that they are plausible.

Another consideration for interpolation is how much missing data are to be interpolated. If a trial contains too many missing data points, the interpolated results might not be trustworthy. PupEyes supports setting the maximum proportion of missing values that can be allowed for interpolation. Trials with the proportion of missing values above this threshold will not be interpolated.

Here, we use a linear interpolation with a missing threshold of 40%.
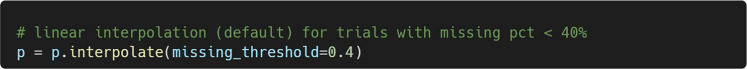


#### Baseline correction

When researchers are interested in task-evoked pupillary responses, it is useful to adjust pupil size relative to a baseline to obtain a measure of pupil size change. The baseline value is typically defined as the mean pupil size during a brief period immediately preceding the onset of the critical stimulus. PupEyes offers two commonly used baseline correction methods:

- Subtractive: corrected size = pupil size – baseline
- Divisive: correct size = pupil size/baseline

Mathôt et al. (2018) advised against divisive baseline correction due to its proneness to produce outlier traces. PupEyes, by default, uses the subtractive method.

PupEyes assumes that the baseline data are in the same pandas data frame as the to-be-corrected data. In our example, we use the mean of the last 100 samples of the “fixation” period for baseline correction.
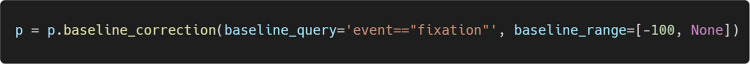


#### Chained operations

The above operations can be chained together to create a preprocessing pipeline, which improves code readability and reduces redundancy.
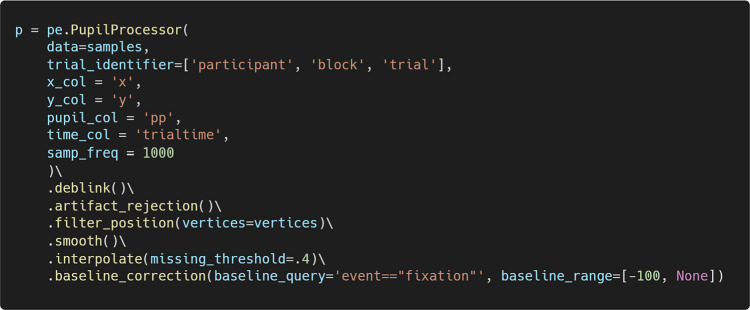


**Fig. 6:**
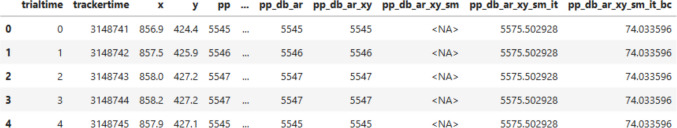
Data after all preprocessing steps

The resulting dataset following all the above preprocessing steps is shown in Fig. 6. A new column is added for each preprocessing step, with the column suffix indicating the specific operation performed. As such, PupEyes preserves all intermediate data in the order in which preprocessing steps are performed. The suffix system is used to reduce the length of column names. As users perform each preprocessing step, the printed messages will indicate which columns are created for each step.

## Quality check using interactive tools

Pupil preprocessing typically involves multiple steps, each controlled by several adjustable parameters. Therefore, it is important to inspect the data to ensure that they are being cleaned properly and to adjust the preprocessing pipeline if necessary. To support this, PupEyes provides several interactive diagnostic tools that allow users to thoroughly assess preprocessing quality.

### Pupil Viewer

Pupil Viewer allows users to interactively inspect processed pupil traces at each step of the preprocessing pipeline. To run Pupil Viewer, pass the PupilProcessor object to PupilViewer(). Users can optionally provide a column name to pass to the hue argument to visualize trial components in different colors.
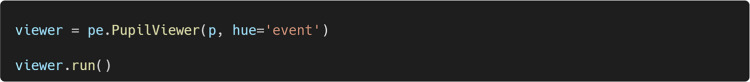


**Fig. 7:**
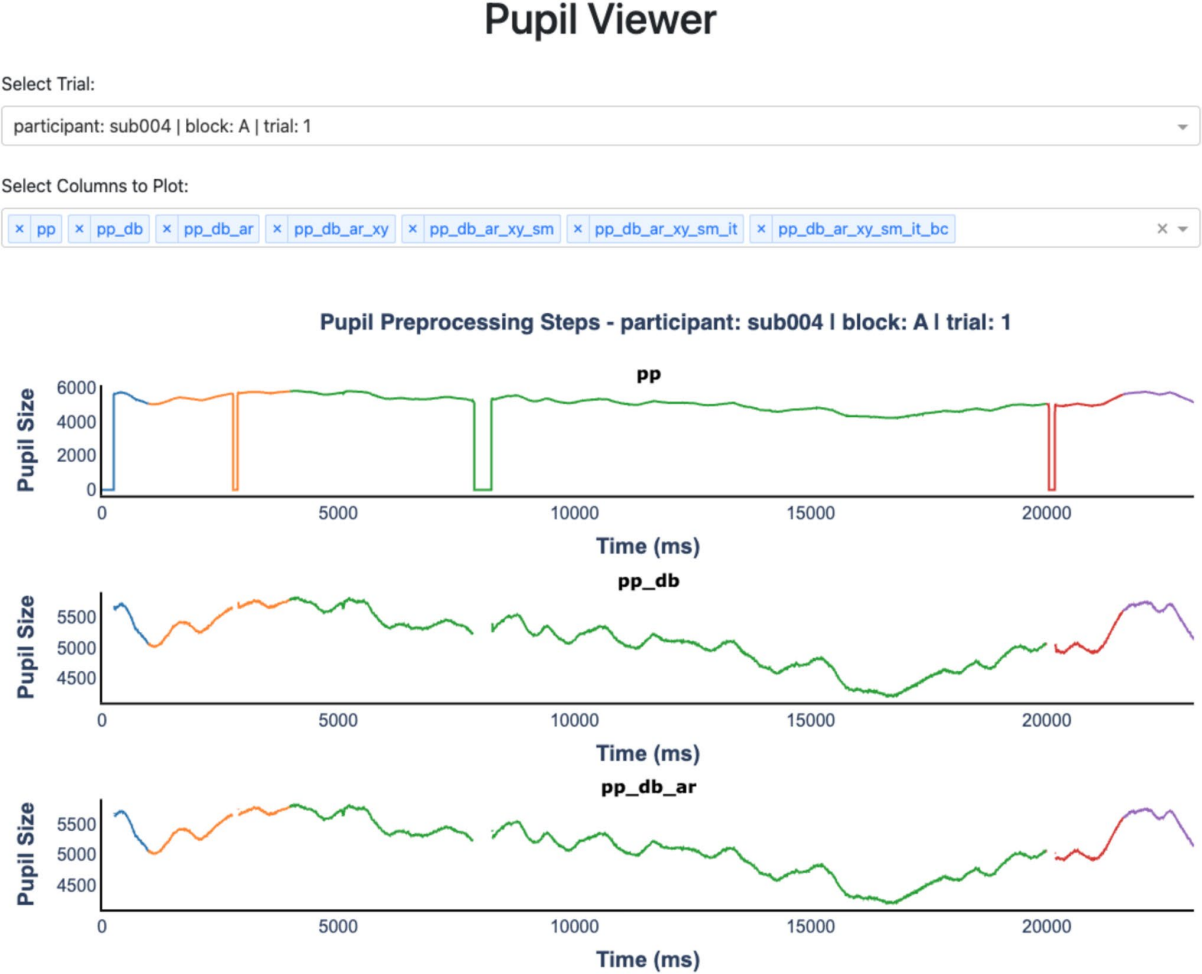
A screenshot of Pupil Viewer

Figure 7 shows a screenshot of the launched Pupil Viewer. Users can use interactive features such as hovering over data points, dragging, and zooming in and out to explore their data. A dropdown menu allows users to select any trial for inspection. Example uses of Pupil Viewer include verifying whether blinks are effectively removed, ensuring that extreme pupil sizes are properly excluded, ensuring that smoothing is applied appropriately, verifying that interpolation has worked as expected, and confirming that baseline correction is correctly applied.

### Pupil foreshortening surface

**Fig. 8:**
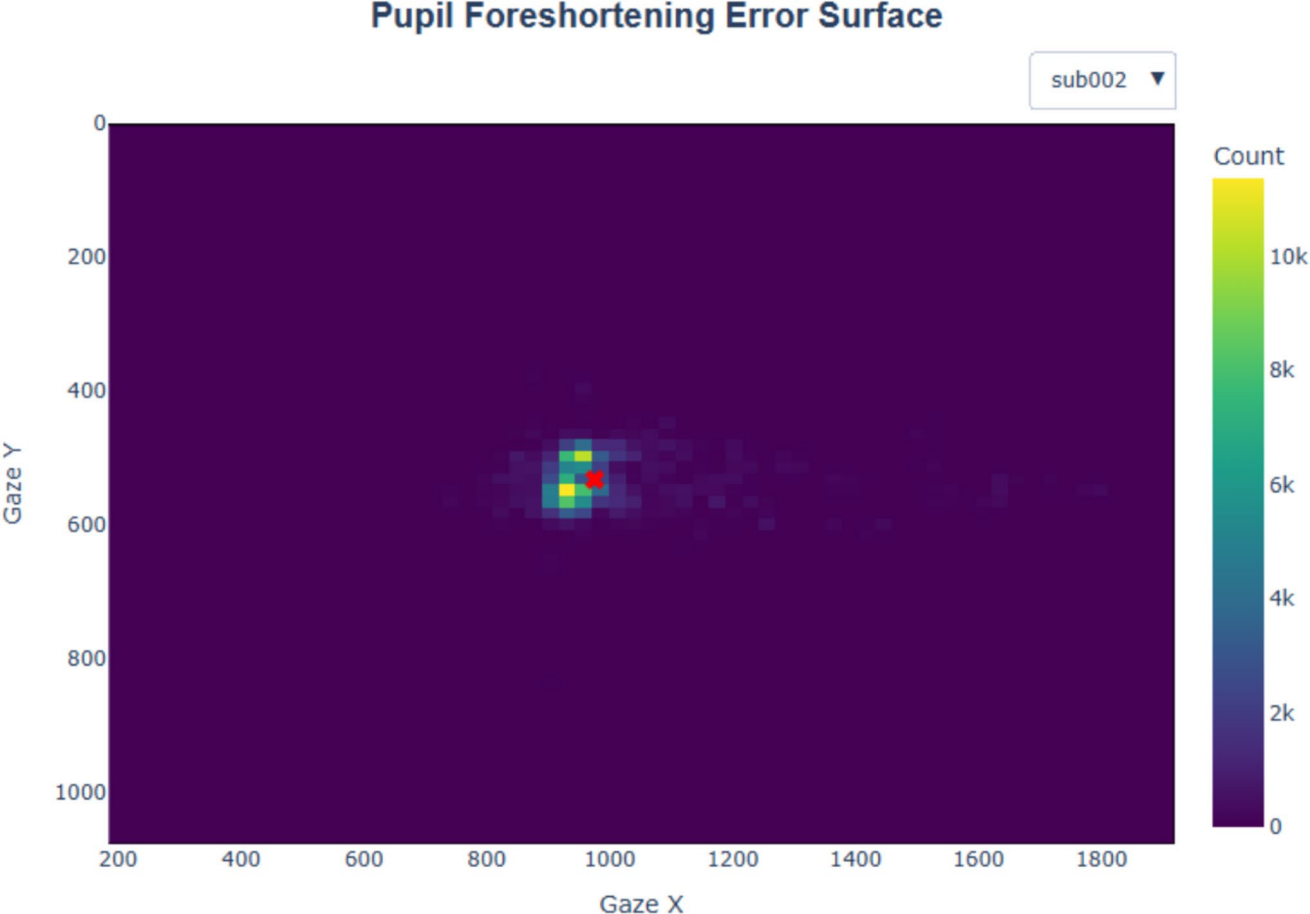
A pupil foreshortening surface plot showing a 2D histogram of recorded samples as a function of gaze coordinates


The plot_pupil_surface() function generates a two-dimensional (2D) histogram showing the frequency of recorded samples as a function of gaze coordinates (see Fig. 8). The red cross indicates the average gaze position. This visualization allows users to assess the spatial spread of gaze. In addition, plot_pupil_surface() supports an alternative visualization (not shown here) showing the *average pupil size* as a function of gaze coordinates (Kinley & Levy, 2021). This mode is activated when passing the argument plot_type=’size’. Together, these two visualizations help researchers assess the spatial spread of gaze and the extent of PFE in their data.

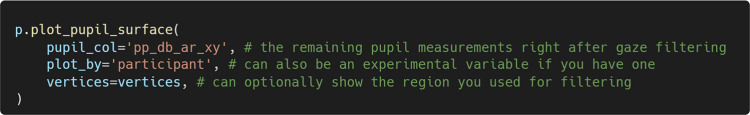


### Baseline histogram

**Fig. 9:**
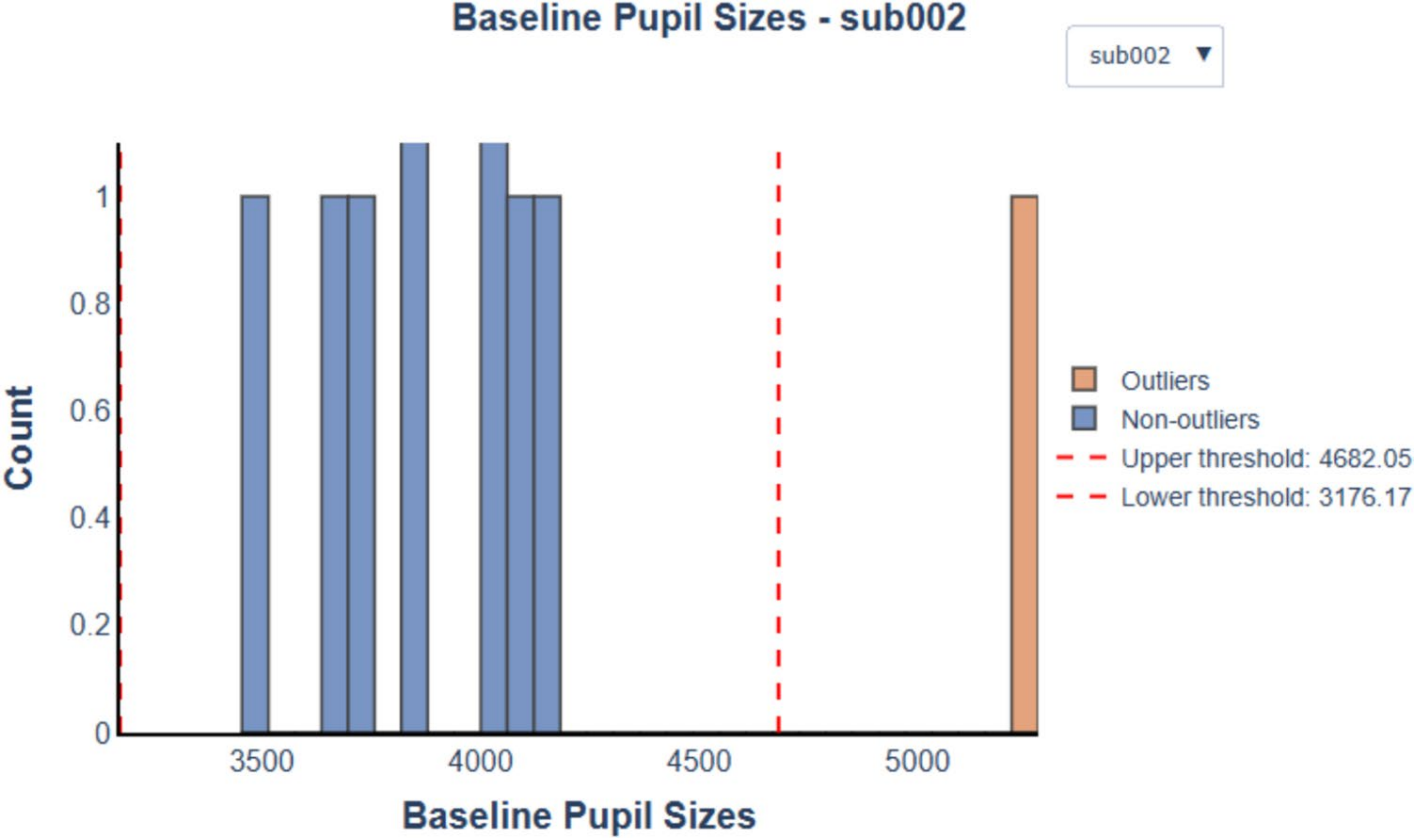
A baseline histogram generated using check_baseline_outliers() for an example dataset containing 10 trials. Outlier thresholds are annotated as dashed lines


As previously noted, we used the mean of the last 100 samples of the fixation period for baseline correction. However, if any of these baseline values are distorted, they may introduce downstream effects in subsequent analyses. To address this, PupEyes includes the check_baseline_outliers() method, which generates a histogram of baseline pupil values and highlights suspected outliers (see Fig. 9). The outlier thresholds are defined as [Median – *n* × MAD, Median + *n* × MAD], where *n* is set to 4 by default. Users can choose to apply outlier detection by group (e.g., participant).

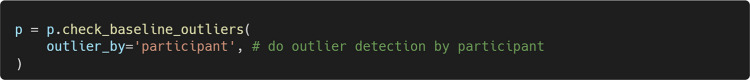


It is important to note that the identified outliers are only suggestions. Mathôt et al. (2018) cautioned against relying on a fixed criterion for detecting baseline outliers, as baseline pupil size distributions can vary considerably across datasets, making a single threshold potentially inappropriate for all situations. Potential outliers may appear as a small cluster of values that deviate from the main distribution in the histogram. The interactive histogram enables users to closely examine the distribution of baseline values and make informed decisions about whether outlier removal is warranted.

### Trace spaghetti plot

**Fig. 10:**
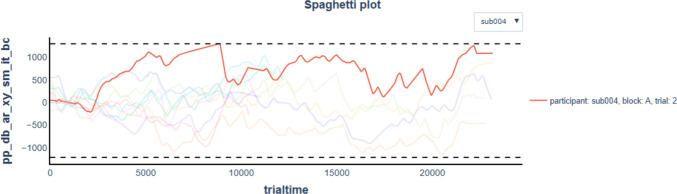
A trace spaghetti plot produced using check_trace_outliers() based on an example dataset containing 10 trials. Outlier thresholds are annotated as dashed lines


In a trace spaghetti plot, all pupil traces are plotted as a function of time (see Fig. 10). High-quality data typically appear as a dense tangle of lines. PupEyes highlights suspected outlier traces based on a distance-based threshold. For each pupil trace, the maximum absolute distance from the grand mean pupil size is computed. Then, it computes the median and MAD of these maximum distances. The outlier threshold is defined as [Grand mean − Median deviation − *n* × MAD, Grand mean + Median deviation + *n* × MAD], where *n* is set to 4 by default. Any trace that exceeds this range at any point in time is flagged as a potential outlier.

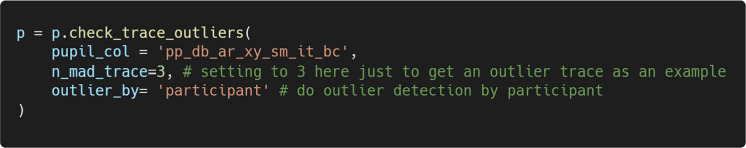


Again, these flagged traces are suggestions only; researchers should make informed decisions based on the characteristics of their specific dataset. Mathôt and Vilotijević (2022) note two indicators of poor-quality data:

- Traces with a sharp downward spike often reflect blinks or other recording artifacts that were not successfully removed.
- Traces that lie well above others or those that begin at zero but sharply rise (within < 200 ms) above others typically indicate abnormally small baseline pupil sizes, which, in turn, produce artificially inflated baseline-corrected pupil sizes.

With the interactive trace spaghetti plot, users should be able to spot these problematic traces if they are not already highlighted as outliers. As will be introduced in the next section, PupEyes allows users to override default outlier detection results and gives users total control over trial exclusion.

## Defining trial exclusion criteria

**Fig. 11:**
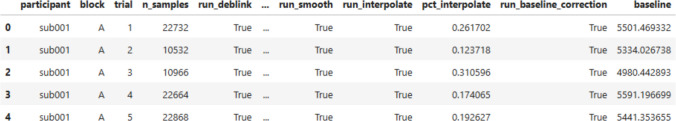
Summary of preprocessing for each trial, returned by summary()


The summary() method returns a pandas data frame that provides an overview of the preprocessing steps applied to the data (see Fig. 11). This summary also includes information about trials flagged as outliers by the check_baseline_outliers() and check_trace_outliers() methods, along with the corresponding outlier thresholds.

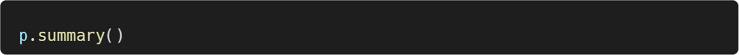


**Fig. 12:**
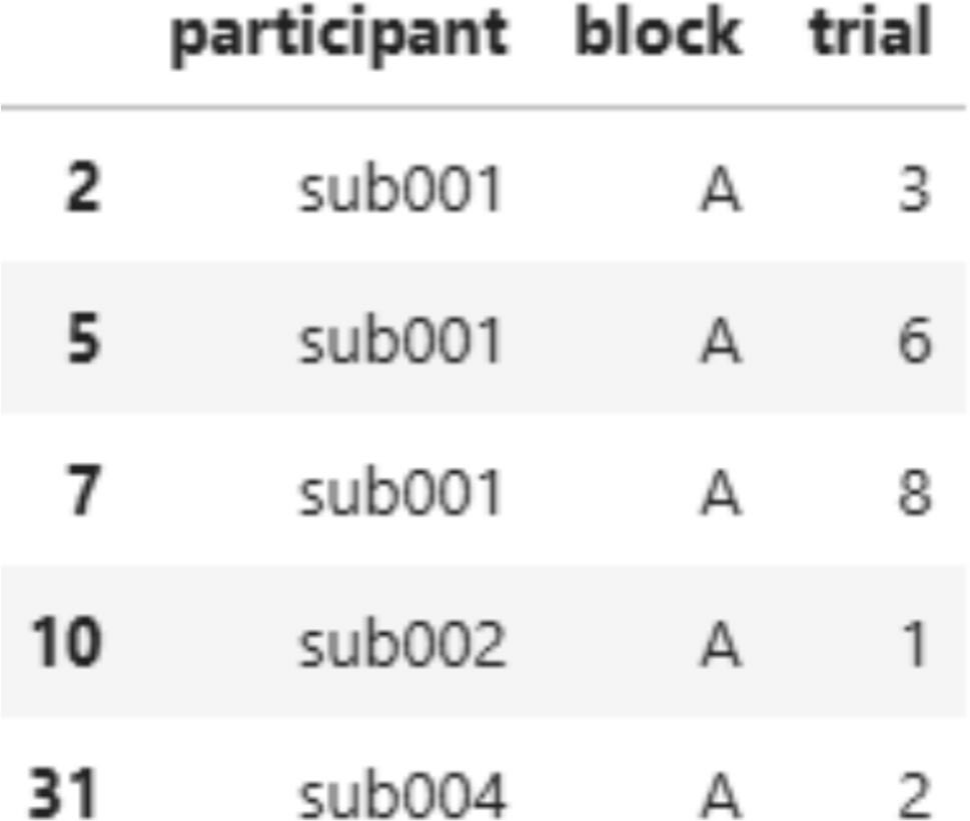
A data frame showing to-be-excluded trials, obtained by subsetting the overall data frame returned by summary()


A primary use of summary() is to develop trial exclusion criteria. For example, researchers may choose to exclude any trial that failed a preprocessing step or was flagged as an outlier. They can do so by using pandas indexing operations to get a data frame of trials to be excluded (see Fig. 12). The numerical summaries allow for the creation of more refined exclusion criteria.

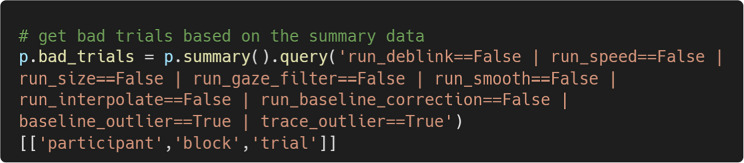


After examining the data using PupEyes’ interactive diagnostic tools, researchers may determine that certain trials flagged as outliers should be retained. These adjustments can be made using standard pandas operations.
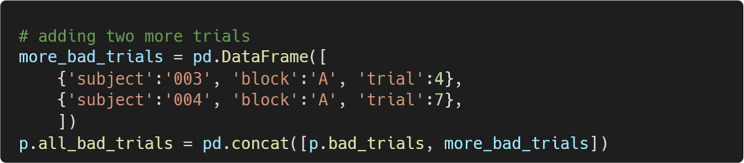


Once a final data frame of trials to exclude has been created, the validate_trials() method can be used to apply these exclusions. To use it, simply pass the data frame of invalid trials as an argument.
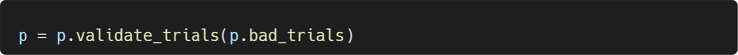


**Fig. 13:**
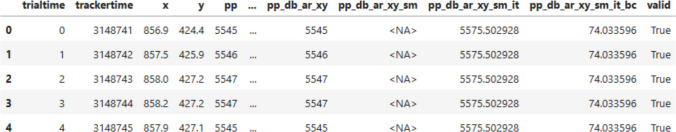
A "valid" column is added to the pupil size data to indicate the validity of each sample

The method adds a Boolean column named “valid” to the pupil size data, indicating whether a sample (or a trial) should be excluded (see Fig. 13). Having a single column indicating the validity of data is convenient for excluding samples and calculating the proportion of valid samples.

### Tidying up

At this stage, users typically want to generate a final, lightweight version of the dataset for further analysis. Since PupEyes uses the pandas data structure, users can apply any standard pandas operations to manipulate their data. Below is an example demonstrating pandas operations used to produce a clean and lightweight dataset. While the specific steps may vary depending on the structure of the researchers’ own data, this example illustrates the flexibility for custom data processing. Users may refer to the executable notebooks (https://pupeyes.readthedocs.io/) to inspect these operations.
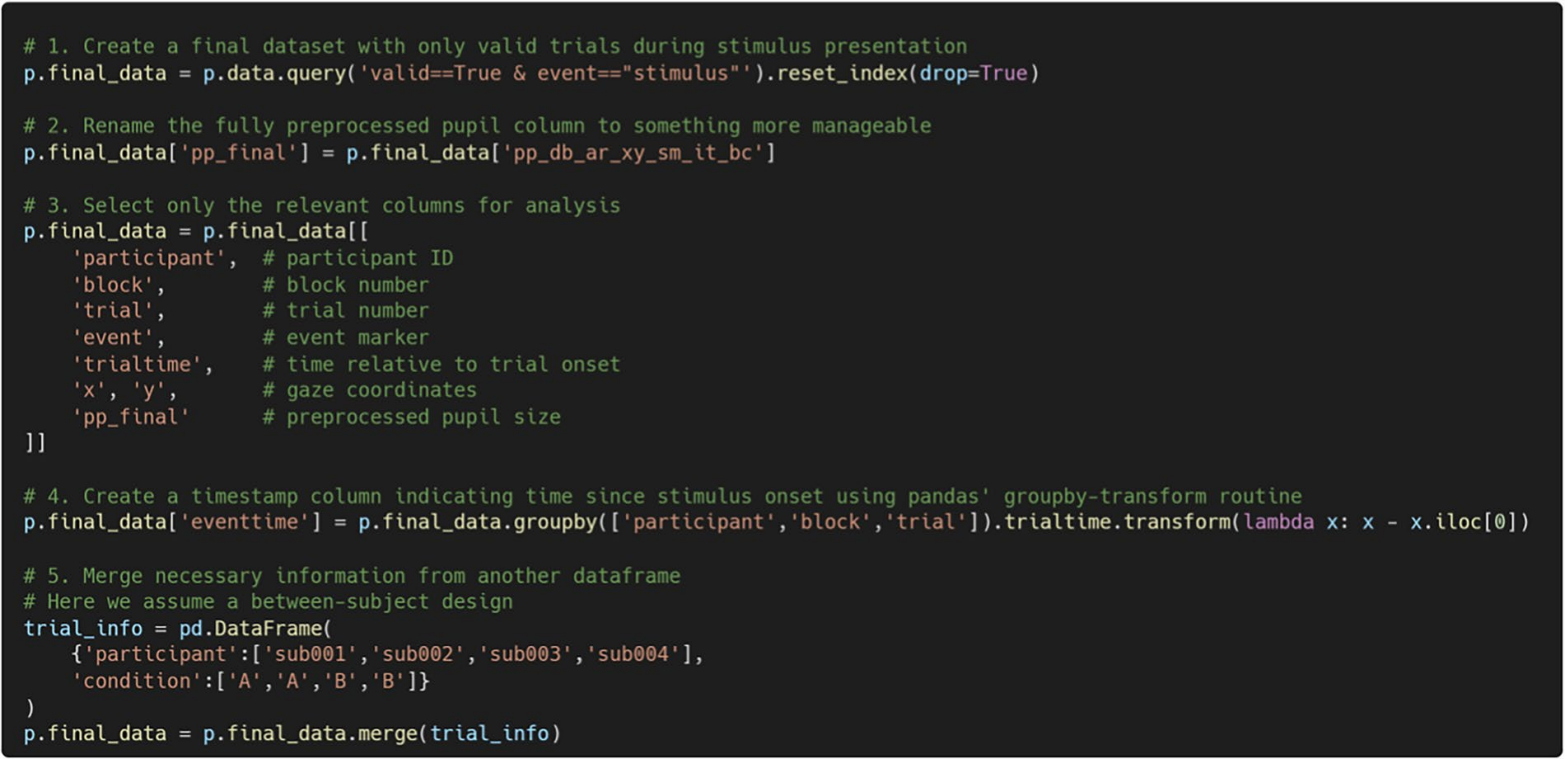


## Statistical analysis of pupil size data

PupEyes focuses on preprocessing—preparing pupil size data in an analysis-ready format. In some sense, the role of PupEyes ends once the data are cleaned and ready for statistical analyses. Given the diverse options for analyzing pupil data, it would be impractical to implement all statistical methods in PupEyes. Existing Python packages such as statsmodels (Seabold & Perktold, 2010) and MNE (Gramfort et al., 2013) already offer robust analysis capabilities, so duplicating those efforts is not necessary. Users may also choose to export the preprocessed data and conduct statistical analysis in a different environment, such as R. That said, because PupEyes is based on pandas, the preprocessed data are fully compatible with other statistical analysis packages in Python. To demonstrate this compatibility, we provide examples showing how PupEyes can be integrated with external packages to conduct basic statistical analyses: https://pupeyes.readthedocs.io/en/latest/pupil_stats.html.

### Working with fixation data

In addition to pupil data, PupEyes includes (1) a Fixation Viewer for interactively visualizing fixation data, (2) an AOI drawer tool for defining areas of interest (AOIs), and (3) functions for computing basic AOI-based metrics.

#### The Fixation Viewer

**Fig. 14:**
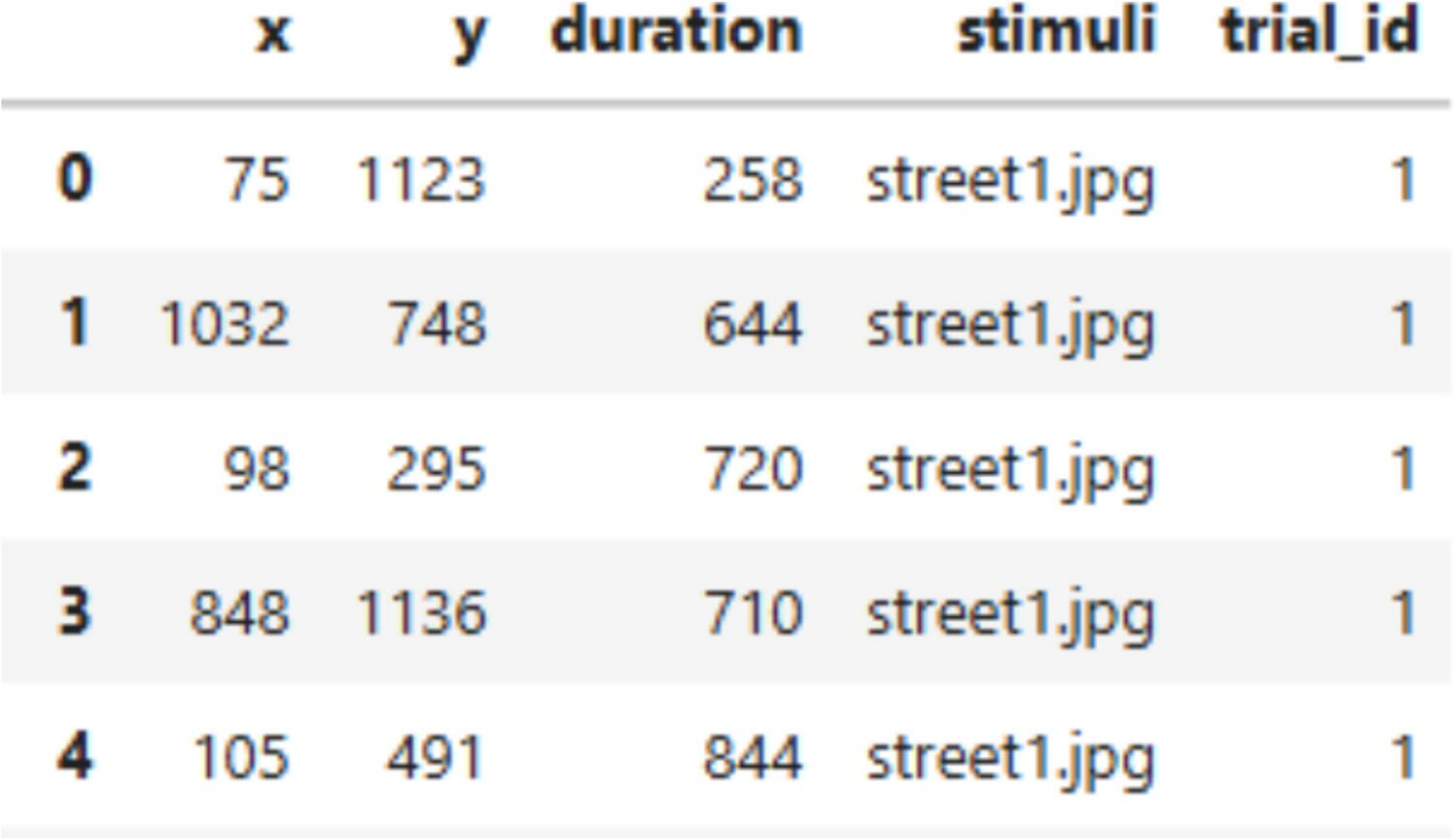
An example dataset of fixations

Figure 14 shows an example dataset that can be used in FixationViewer. In this dataset, each row represents one fixation. The minimum required fields are the *x* and *y* coordinates of each fixation and a trial identifier that identifies unique sets of fixations (e.g., trial_id). FixationViewer can work with data collected from any eye-tracker as long as these columns are provided. Stimulus information and fixation duration are optional.

To use AOI-based visualizations, users must define AOIs in advance. AOIs can be specified as a Python dictionary in which each key–value pair consists of an AOI name and a list of vertices. The last point must repeat the first to close the polygon. For example:
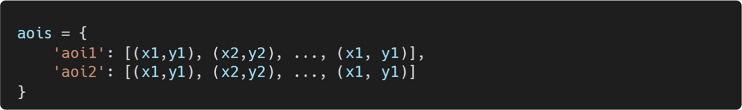


If there are different AOIs for each trial, they can also be specified using a nested dictionary structure in the following format:
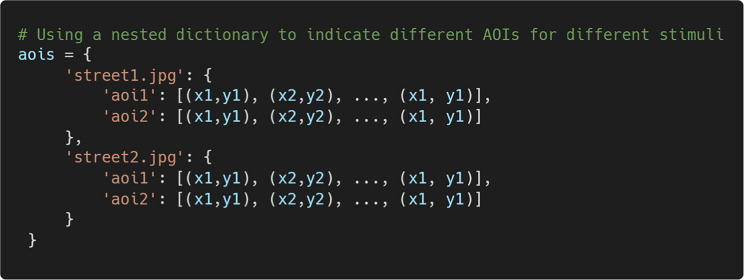


Once all necessary information is provided, one can initialize the FixationViewer by passing the correct arguments. Users can use the set_aois() method to add the AOIs.
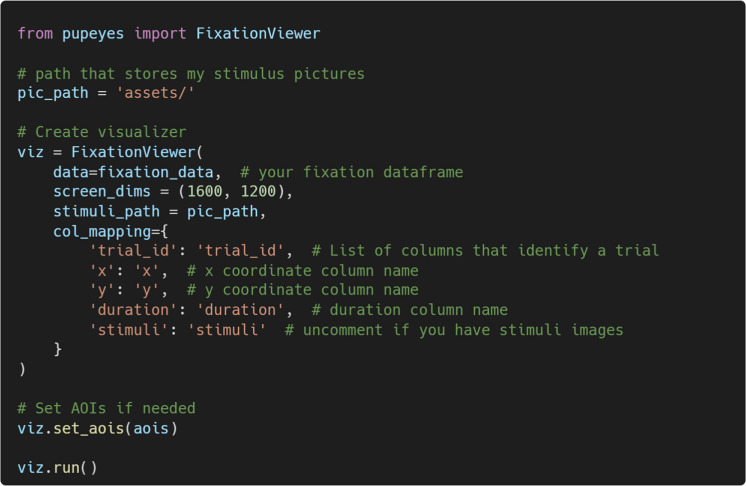


**Fig. 15:**
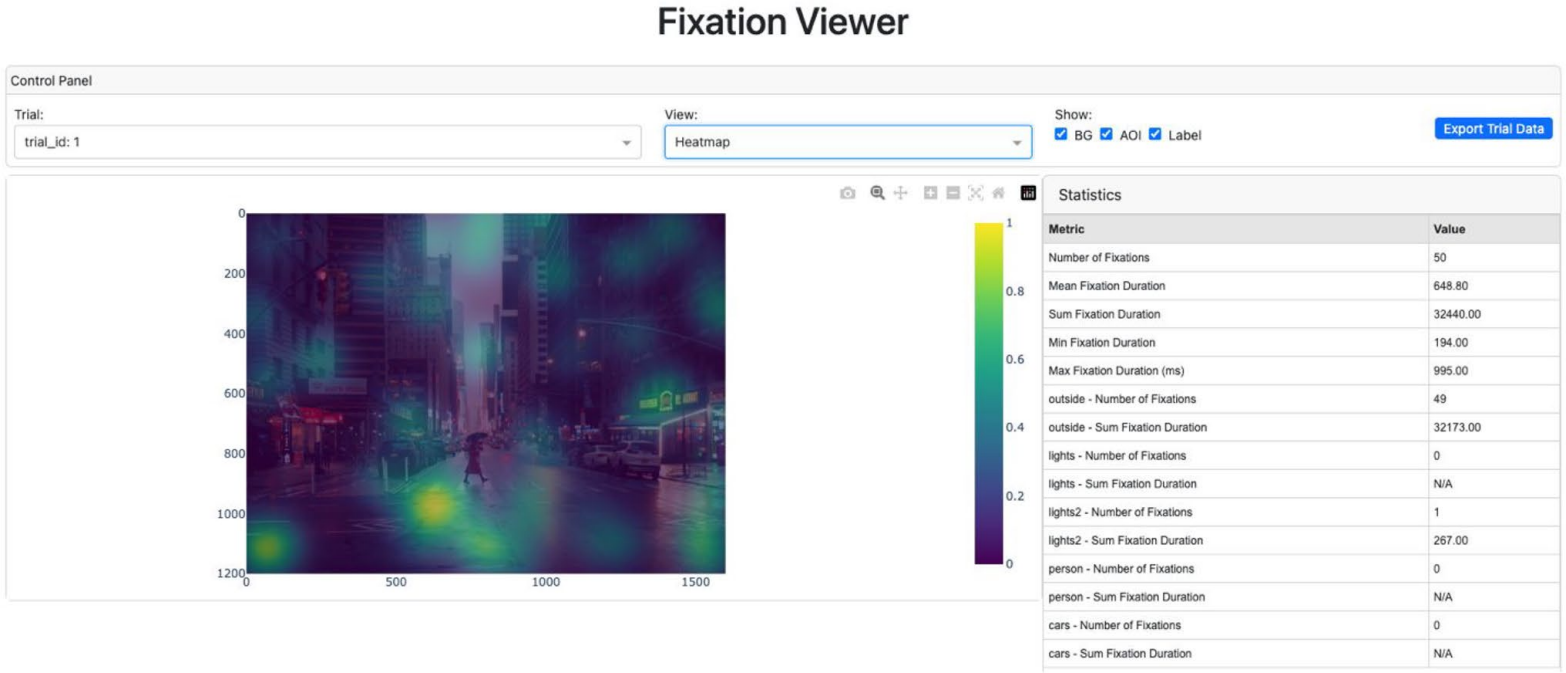
A screenshot of FixationViewer

Figure 15 shows a screenshot of the FixationViewer. It provides three visualizations:

1. A playback of the scanpath,
2. A fixation density map (i.e., heatmap), and
3. An AOI map.

These visualizations can be especially useful for understanding eye movement patterns prior to conducting statistical analyses. Additionally, FixationViewer() displays basic fixation metrics such as fixation count and duration. Finally, there is also an option to export the associated trial data to a text file.

#### The AOI drawer

**Fig. 16:**
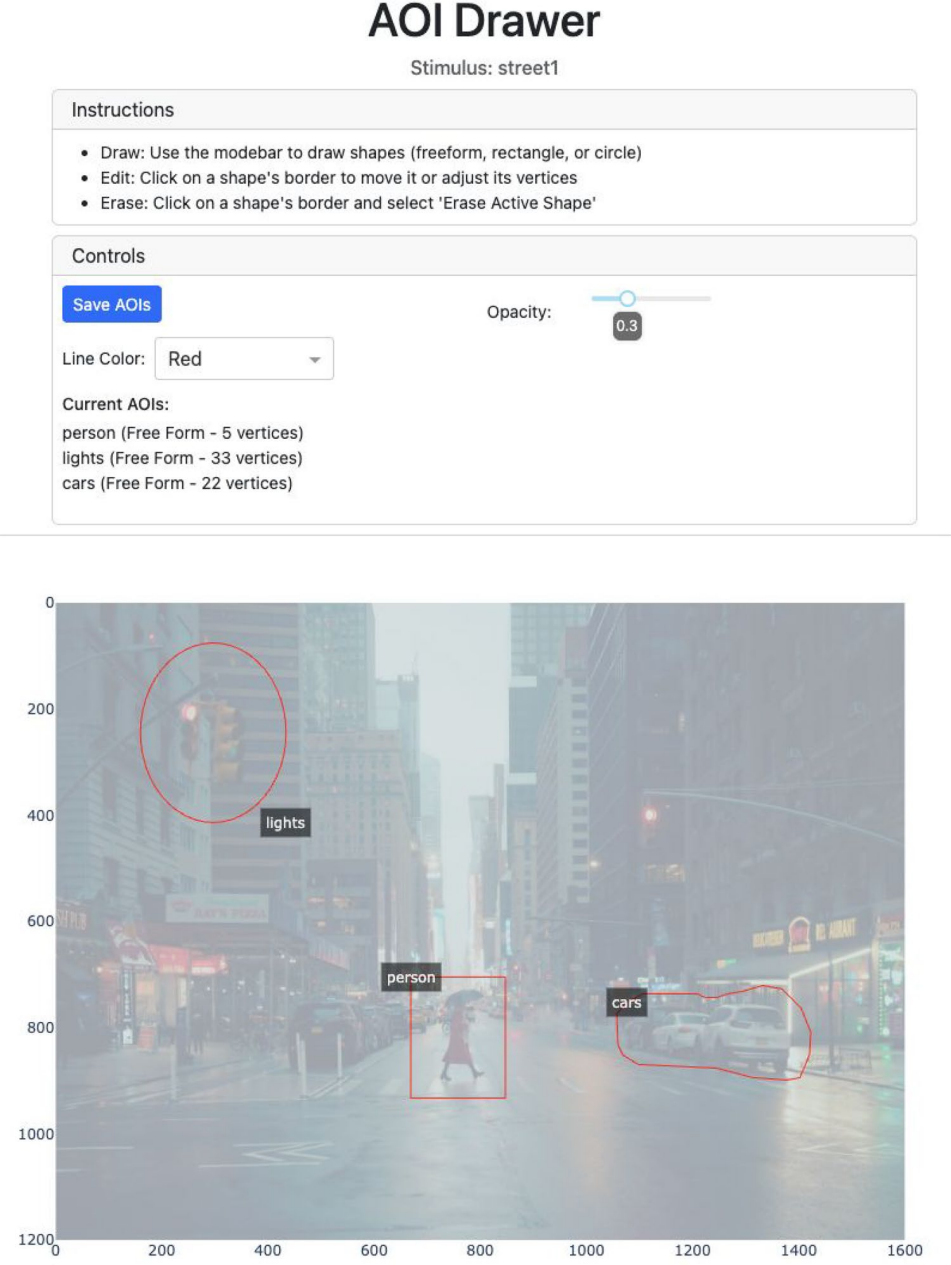
A screenshot of AOI Drawer

Manually specifying the coordinates of AOIs can be cumbersome. As such, PupEyes includes an AOIDrawer tool that allows users to draw AOIs. Figure 16 shows a screenshot of the AOI Drawer interface. Users can create AOIs using freeform shapes, rectangles, or circles, and can edit existing AOIs to adjust their size or shape. Once defined, AOIs are saved as a .json file and can be loaded and used in other PupEyes functions, including FixationViewer.

#### Computing AOI-based measures

To illustrate how the defined AOIs can be used in subsequent analyses, we will conduct a simple AOI-based analysis by assigning each fixation to an AOI and computing basic AOI-related metrics. The compute_aoi_statistics() function calculates summary measures based on AOI assignment, given the fixation *x* and *y* coordinates, defined AOIs, and fixation durations.
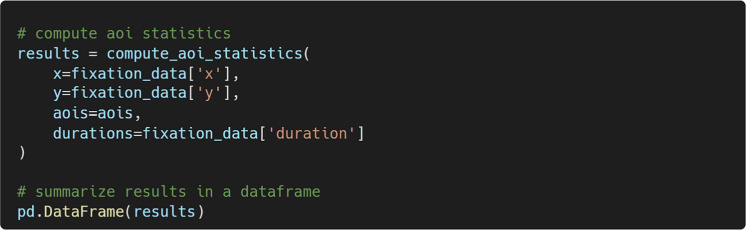


**Fig. 17:**
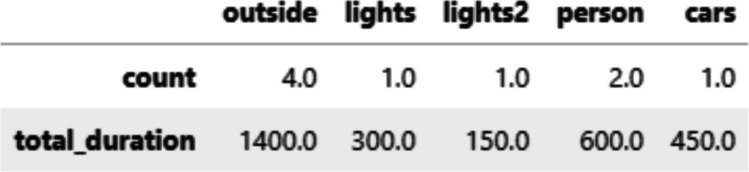
AOI-based metrics calculated by PupEyes

For each AOI, PupEyes calculates the number of fixations and the total duration of fixations (see Fig. 17).

## Discussion

We have introduced PupEyes, an open-source Python package for preprocessing and visualizing pupil size and fixation data. The pupil preprocessing pipeline, informed by current best practices, ensures that pupil size data are handled in a principled, transparent, and reproducible manner. PupEyes’ interactive visualizations enable researchers to explore their data, inspect individual trials, and detect potential outliers. Finally, PupEyes uses the pandas data structure, allowing users to leverage Python’s powerful and flexible tools for further data analysis and manipulation.

PupEyes is useful not only for research but also for teaching and training. First, all intermediate steps are preserved during pupil preprocessing and can be visualized using the PupilViewer. This makes it easy to demonstrate what each preprocessing step does and how different pipelines can affect the final results. Second, the Fixation Viewer provides intuitive visualizations of eye movement data. The growing use of open-source tools for eye-tracking means that trainees often lack access to visualizations commonly included in commercial software. As a result, they frequently work with eye-tracking data in raw tabular form (much like reaction time data), which can obscure the dynamic nature of eye movements. Easy access to visualizations helps trainees better understand their data, stay engaged, and gain new insights. Finally, the intuitive API and detailed online tutorials offered by PupEyes provide a gentle introduction to using Python for data analysis.

### Comparison to other packages

Table 1 presents a comparison between PupEyes and similar packages. The pypillometry package is an open-source Python library for pupil size preprocessing (Mittner, 2020). Due to similarities in language and goals, the development of PupEyes was heavily inspired by pypillometry. Both packages adopt a pipeline-based approach that supports common preprocessing operations such as deblinking, smoothing, and interpolation (though the specific implementations differ). Both also include a summary method to report preprocessing steps. While pypillometry offers some basic visualizations of pupil size data, PupEyes provides a more comprehensive set of interactive visualizations for both pupil and fixation data. Furthermore, PupEyes includes convenient functions for reading raw EyeLink data, and, in addition to pupil size preprocessing, PupEyes supports visualizing fixation data and computing AOI-based metrics. On the other hand, a unique feature of pypillometry is its support for modeling the tonic and phasic components of the pupil signal, a feature currently not available in PupEyes.

**Table 1:** Comparison between PupEyes and several other eye-tracking analysis packages

	PupEyes	pypillometry	PyTrack	gazeR	pupillometry	pupillometryR	CHAP	PuPl
Language	Python	Python	Python	R	R	R	MATLAB	MATLAB
Tracker-specific support	EyeLink, Tobii	None	EyeLink, Tobii, SMI	EyeLink, Tobii	EyeLink, Tobii, SMI	None	EyeLink, EyeTribe, Tobii, ASL	EyeLink, Tobii, EyeTribe, SMI
Customizable pipeline	Yes	Yes	No	Yes	Yes	Yes	No	Yes
Deblink	Yes	Yes	Yes	Yes	Yes	Yes	Yes	Yes
Artifact rejection	Yes	No	No	Yes	Yes	No	Yes	No
Position filtering	Yes	No	No	No	No	No	No	Yes
Smoothing	Yes	Yes	No	Yes	Yes	Yes	Yes	Yes
Interpolation	Yes	Yes	Yes	Yes	Yes	Yes	Yes	Yes
Baseline correction	Yes	Yes	No	Yes	Yes	Yes	Yes	Yes
Resampling	Yes	Yes	Yes	Yes	Yes	Yes	Yes	Yes
Interactive diagnostic plots	Yes	Limited	No	No	No	No	No	Yes
Fixation visualization	Yes	No	Yes	No	No	No	No	No
AOI metrics	Yes	No	Yes	Yes	No	No	No	No
Advanced statistical methods	No	Yes	Yes	No	No	Yes	Yes	No

PyTrack is another open-source Python library for analyzing and visualizing eye-tracking data (Ghose et al., 2020). It allows users to extract multiple eye-tracking measures, visualize eye movement patterns, and perform basic statistical analyses. However, its pupil preprocessing pipeline is hard-coded into the codebase, so users cannot adjust preprocessing steps to suit their specific needs. It also returns a fixed set of pupil size measures, potentially limiting analysis options. Finally, PyTrack does not include interactive visualizations for diagnostic purposes.

Several open-source R packages have also been developed for processing gaze position and/or pupil size data, including GazeR (Geller et al., 2020), pupillometry (Tsukahara, 2022), and pupillometryR (Forbes, 2020). Although varying in implementation details, these packages support common pupil preprocessing steps. None of these packages offers interactive visualizations of pupil and fixation data.

Finally, CHAP (Hershman et al., 2019) and PuPl (Kinley & Levy, 2021) are MATLAB-based packages for pupil size analysis. CHAP is a GUI-based tool that supports data import, pupil preprocessing, and statistical analysis. While convenient, its GUI-only design may limit computational reproducibility and analysis options. PuPl, on the other hand, offers a comprehensive set of preprocessing functions with interactive diagnostic plots. Notably, PuPl allows users to export GUI actions as scripts, enabling more experienced users to work programmatically while maintaining the advantages of a GUI. However, neither CHAP nor PuPl is designed to work with both pupil size and fixation data within a single library.

Although PupEyes has unique features compared to existing packages, it is not designed to replace the other packages. Rather, it is designed to work *with* them. For example, pupil size data preprocessed with PupEyes may be used in the pypillometry package to model tonic and phasic components. Processed data can also be easily exported to R or other environments for further analysis. Moreover, PupEyes can be used solely for extracting gaze samples or visualizing fixation patterns. Then, users may switch to another package (or another computing environment) to conduct further analyses. This flexibility allows users to integrate PupEyes into their existing workflows based on specific research needs.

### Future plans

As best practices for pupil size preprocessing continue to evolve, PupEyes should adapt accordingly to ensure its functionalities remain current. Another potential direction is to develop task-specific functionalities. For example, in reading research, researchers often need to compute word-based metrics such as gaze duration and total viewing time (Rayner, 1998). In visual search paradigms, researchers are often interested in the landing position of the initial saccade (Gaspelin & Luck, 2018). Finally, PupEyes currently supports reading raw data collected from EyeLink and Tobii eye-trackers, and we plan to include support for data formats collected with other eye-tracking systems in the future.

As an open-source project, PupEyes welcomes contributions from the research community. We encourage users to suggest new features, report bugs, improve documentation, and contribute code to improve PupEyes. Visit our GitHub page (https://github.com/HanZhang-psych/pupeyes) to learn more.

We hope PupEyes makes pupil and eye movement data analysis more accessible, transparent, and fun for the eye-tracking community.

## Data Availability

Data for this paper can be found at https://github.com/HanZhang-psych/pupeyes/tree/main/docs/data

## Code availability

The software described in this paper is available at https://github.com/HanZhang-psych/pupeyes.
